# Macrophage Meets the Circadian Clock: Implication of the Circadian Clock in the Role of Macrophages in Acute Lower Respiratory Tract Infection

**DOI:** 10.3389/fcimb.2022.826738

**Published:** 2022-02-23

**Authors:** Ken Shirato, Shogo Sato

**Affiliations:** ^1^Department of Molecular Predictive Medicine and Sport Science, Kyorin University School of Medicine, Mitaka, Japan; ^2^Center for Biological Clocks Research, Department of Biology, Texas A&M University, College Station, TX, United States

**Keywords:** circadian rhythm, circadian clock, macrophage, inflammation, acute lower respiratory tract infection, phagocytosis, pro-inflammatory response, efferocytosis

## Abstract

The circadian rhythm is a biological system that creates daily variations of physiology and behavior with a 24-h cycle, which is precisely controlled by the molecular circadian clock. The circadian clock dominates temporal activity of physiological homeostasis at the molecular level, including endocrine secretion, metabolic, immune response, coupled with extrinsic environmental cues (*e.g.*, light/dark cycles) and behavioral cues (*e.g.*, sleep/wake cycles and feeding/fasting cycles). The other side of the clock is that the misaligned circadian rhythm contributes to the onset of a variety of diseases, such as cancer, metabolic diseases, and cardiovascular diseases, the acceleration of aging, and the development of systemic inflammation. The role played by macrophages is a key mediator between circadian disruption and systemic inflammation. At the molecular level, macrophage functions are under the direct control of the circadian clock, and thus the circadian misalignment remodels the phenotype of macrophages toward a ‘killer’ mode. Remarkably, the inflammatory macrophages induce systemic and chronic inflammation, leading to the development of inflammatory diseases and the dampened immune defensive machinery against infectious diseases such as COVID-19. Here, we discuss how the circadian clock regulates macrophage immune functions and provide the potential risk of misaligned circadian rhythms against inflammatory and infectious diseases.

## Introduction

Acute lower respiratory tract infections (LRTIs) remain the most lethal communicable diseases by causing life-threatening pneumonia. Lung-resident alveolar macrophages play a pivotal role in the clearance of airborne pathogenic microorganisms, including bacteria, fungi, and viruses. In fact, alveolar macrophages have remarkable phagocytic activity owing to the high expression of receptors that recognize bacteria and fungi (Steele et al., 2003; Arredouani et al., 2004; Steele et al., 2005; Arredouani et al., 2006; Sharif et al., 2013; Bharat et al., 2016; Mitsi et al., 2018). Prompt and sufficient provocation of the initial pro-inflammatory responses is also indispensable to facilitate further mobilization and activation of peripheral phagocytes such as neutrophils and monocytes in infectious foci (Branger et al., 2004; Knapp et al., 2004; Albiger et al., 2007; Boyd et al., 2012; Sánchez-Tarjuelo et al., 2020). In contrast, although alveolar macrophages are not able to eliminate the virus directly, they assist alleviate pneumonia by removing the epithelial and inflammatory cells that caused apoptotic cell death as a result of the anti-viral response (Seitz et al., 2007; Schneider et al., 2014a; Fujimori et al., 2015; Grabiec et al., 2017; Mohning et al., 2018). Thus, failure of these protective actions leads to excessive and uncontrolled activation of peripheral monocyte-derived inflammatory macrophages, exacerbating pneumonia from mild to life-threatening. Essentially, we summarize the protective roles of alveolar macrophages on acute LRTIs *via* phagocytosis, pro-inflammatory responses, and efferocytosis.

Macrophage functions are reprogramed by cues derived from our daily lifestyle. Diet (*i.e.*, nutrition), physical exercise, and aging progression has been revealed to modulate the phenotype of macrophages, defining systemic inflammatory status (Olefsky and Glass, 2010; Dall’Asta et al., 2012; Kizaki et al., 2012; Linehan and Fitzgerald, 2015; Davanso et al., 2020; Sitlinger et al., 2020; De Maeyer and Chambers, 2021; Duong et al., 2021). It has begun to be accepted that both the quantity and quality of immune cells including monocytes/macrophages exhibit a robust daily oscillation (Curtis et al., 2014; Leach and Suzuki, 2020; Shimba and Ikuta, 2020; Waggoner, 2020; Cermakian et al., 2021). The accumulating evidence illustrates the molecular mechanisms underlying the biological clock oscillators regulate temporal diversity of immune activities in accordance with cyclic environmental cues (*e.g.*, light-dark cycles and temperature fluctuation) and behavioral rhythms (*e.g.*, sleep-wake cycles, feeding-fasting cycles), which orchestrates the temporal regulation of immune homeostasis. In general, corresponding to the activity cycles, the immune system is poised for pathogenic attacks in the morning while it undergoes tissue repair and regeneration at night. This dynamic time-of-day variation of immune activity results in different responses to pathogen infection and thereby infectious morbidity and mortality and post-surgery recovery (Kvaslerud et al., 2010; Knutson and von Schantz, 2018; Sengupta et al., 2019). Thus, understanding of time-of-day-dependent functions of immune cells such as macrophages is of importance to directly complement personalized health development and further develop chronobiology- and chronopharmacology-based infectious disease treatment. Here we discuss and provide a perspective on the implication of the circadian regulation of macrophage functions unique to alveolar macrophages and peripheral monocyte-derived macrophages.

## Circadian Rhythms in Immunity

Macrophage immune functions are adaptive to both internal physiological and external environmental changes. For instance, macrophage metabolic perturbation following nutritional challenge reprograms macrophage activity including a shift of macrophage polarization, secreted cytokines, tissue infiltration (Liu et al., 2020; Wu and Ballantyne, 2020). The relationship between aging and macrophage functions is also well-characterized. The most recent review article has summarized how the onset and progression of diseases, as well as deteriorating health in the elderly, are accompanied by reprogramming of macrophage functions (Duong et al., 2021). Ultimately, macrophage functions, and in particular redox homeostasis, is a potential modulator of life-span and aging progression (Vida et al., 2017), and overall, fine-tuned immune homeostasis not only in macrophages but also in immune cells including myeloid cells directly rejuvenates age-dependent degradation of biological functions represented by remyelination of aged central nervous system (Rawji et al., 2020), bone repair (Vi et al., 2018), and cognitive improvement (Minhas et al., 2021).

The dynamics of macrophage inflammatory signatures also specify temporal diversity of immune activity. Cyclic regulation of immune activity includes daily fluctuation of the number of circulating leukocytes, levels of secreted hormones and cytokines related to the regulation of immune activity, tissue infiltration of macrophages, innate and adaptive immune activities, a temporal gating of cellular responses to endotoxin challenge, and inflammatory signaling pathways and gene programs (Keller et al., 2009; Gibbs et al., 2012; Silver et al., 2012; Curtis et al., 2014; Sato et al., 2014b). The cyclic oscillation of immune functions allows organisms to anticipate daily variation of behavioral activities (*i.e.*, sleep/wake cycles and feeding/fasting cycles) and thereby risks of infection and tissue damage (Haspel et al., 2020). The circadian dynamics of immune activity gates the timing not only for the defense system to the bacterial and viral pathogen but also for tissue repair and regeneration against damage and injury.

The word ‘circadian’ derived from *the Latin* phrase ‘*Circa*’ and ‘*Diem*’ means ‘about a day’. The circadian rhythms refer to biological rhythms with a cycle of approximately 24-hour corresponding to the Earth’s 24-hour rotation (Bass and Lazar, 2016). Earth rotation around the axis in about 24-hour results in robust daily environmental cycles, including day-night cycles, followed by light-dark cycles and temperature oscillation. Thus, the circadian rhythms can be rephrased as adaptive biological systems to cyclic environmental changes. The circadian rhythms appear at any levels of biological processes from molecular to behavioral levels, including sleep-wake cycles, endocrine secretion, metabolism, and immunity (Green et al., 2008; Curtis et al., 2014; Panda, 2016; Top and Young, 2017). These biological rhythms need to be well aligned and harmonized with external environmental cycles for health maintenance – *i.e.*, the disruption of the circadian rhythms is critically detrimental for physical health conditions (Knutsson, 2003; Fonken et al., 2010; Masri and Sassone-Corsi, 2018; Sato and Sassone-Corsi, 2021). The misaligned circadian rhythms have been reported to lead to multiple disease onset and progression, including obesity, type 2 diabetes, cardiovascular diseases, cancer, systemic inflammatory diseases, and psychiatric disorders (Mason et al., 2020; Zhang et al., 2020a; Allada and Bass, 2021). In support of this review, a link exists between clock-controlled functions and lung pathophysiology, and thus the disruption of circadian rhythms leads to lung inflammatory diseases including chronic obstructive pulmonary disease (COPD), asthma, and smoking-stimulated inflammation (Sundar et al., 2015). Chronic disruption of the circadian rhythms is often found in people with shift working and traveling to a different time zone. Epidemiologic studies have revealed a robust relationship between shift workers and many types of diseases, all of which are highly associated with systemic inflammation (Knutsson, 2003; Lyall et al., 2018; Torquati et al., 2018). The mechanisms behind the development of systemic inflammatory diseases elicited by chronic circadian misalignment are associated with the dysregulation of the innate immune system such as the heightened release of proinflammatory cytokines in response to endotoxic shock by lipopolysaccharide (LPS) (Castanon-Cervantes et al., 2010). Specifically, isolated peritoneal macrophages harvested from animals under circadian disruption exhibit a robust response to LPS stimulation. At the molecular level, mouse models for circadian disruption show a dysregulation of inflammatory gene expression in macrophages (discussed in the following section) (Gibbs et al., 2012). These insights directly point to the essential contribution of macrophages to the development of systemic inflammatory status following chronic circadian misalignment.

Cyclic environmental cues are aligned with biological molecular clocks. The synchrony of external cues with the internal time-keeping system is required for the regulation of organismal circadian homeostasis. Light is known to act as the most dominant external time giver called *zeitgeber* in German (LeGates et al., 2014). The light signal is delivered from the retina to the hypothalamic nucleus (SCN) where houses approximately 20 thousand neurons. The SCN molecular clock can be entrained by the light signal from the retina as an external time-givers. The SCN acts as a master pacemaker that coordinates and synchronizes peripheral molecular clock across the entire cells, tissues, and organism. Moreover, circadian behavioral rhythms are orchestrated by a subset of SCN neurons expressing the neuropeptide neuromedin S (NmS). Abolishing the circadian molecular clock of NmS neurons disrupts systemic circadian behavior (Lee et al., 2015; Landgraf et al., 2016). Thus, the loss of appropriate light/dark cycles disrupts circadian biological functions, including irregular temporal oscillation of hormone secretion and gene expression, through the SCN clock.

In addition to the light exposure, the endogenous clock in peripheral tissues is entrained by external environmental and behavioral cues such as sleep-wake cycles, food intake, and physical activity, independent of the SCN clock. For instance, light exposure during the night increases body weight by shifting the time of food intake (Fonken et al., 2010). Moreover, food intake during the biological rest phase contributes to body weight gain, leading to metabolic syndrome (Arble et al., 2009). On the contrary, food intake during the biological active phase prevents mice from high-fat diet-induced obesity and systemic inflammation (Hatori et al., 2012). Thus, circadian timing of behaviors determines health and circadian misalignment of behaviors leads to the onset and progression of diseases and systemic inflammation.

## Circadian Clocks: The Molecular Time-Keeping System

**Figure 1:**
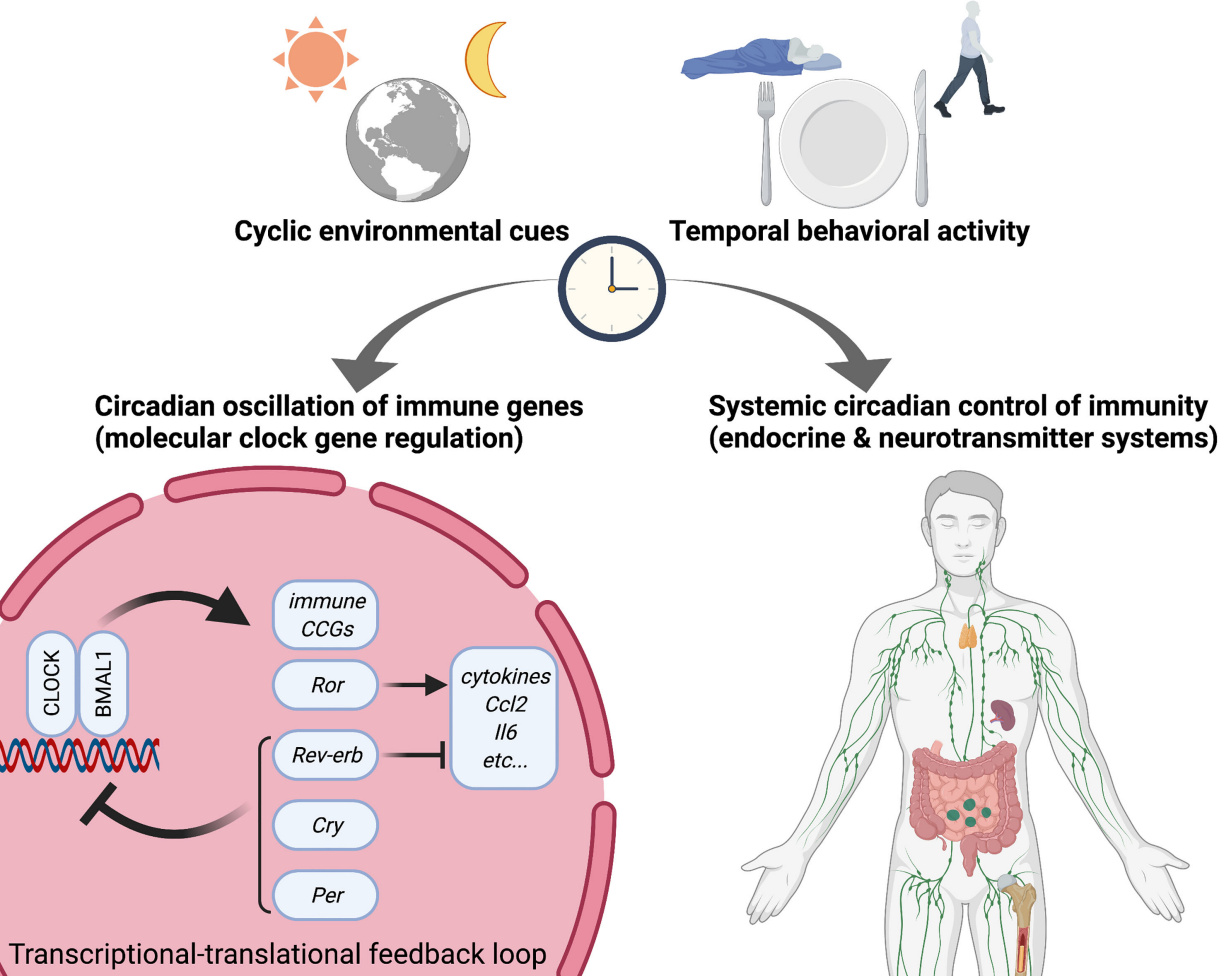
Circadian rhythms in immunity. Circadian rhythms appear in multiple physiological responses including immunity, resulting in circadian gating of temporal diverse activity of immune functions. Not only do circadian endocrine and neurotransmitter mechanisms determine diurnal versus nocturnal immune responses but also molecular circadian clock directly trans-regulates cyclic gene expression related to immune functions such as cytokines and pattern recognition receptors (PRRs). Specifically, several circadian transcription factors (activators) and repressors directly interact with the promoter regions of immune genes. Accordingly, misaligned circadian rhythms due to shift work (social jet-lag) and jet-lag disrupt temporal regulation of immune homeostasis and dynamic temporal balance of pro- and anti-inflammation. CLOCK, Circadian locomotor output cycles kaput; BMAL1, Brain and muscle ARNT-like 1; Ror, retinoic acid-related orphan receptor; Cry, Cryptochrome; Per, Period; CCG, clock-controlled gene.

The biological rhythms are derived from an intrinsic timekeeping system within organisms, the so-called circadian clock system. The timekeeping system allows the organism to correspond to the cyclic environmental changes, thereby engaging the organismal physiology and behavior in responding to the environmental changes at the right time of the day. The basic structure of the molecular clock system has been discovered and illustrated (**Figure 1**): The core circadian clock network is operated by a transcriptional-translational feedback loop (TTFL) comprising both positive and negative arms (Takahashi, 2017). The positive arm includes the circadian transcription factor circadian locomotor output cycles kaput (CLOCK) and brain and muscle ARNT-like 1 (BMAL1). Heterodimerized CLOCK : BMAL1 complexes bind to E-box promoter elements at a series of oscillatory genes called clock-controlled genes to drive their transcription. The negative arm includes the circadian repressor PERIOD (PER) and CRYPTOCHROME (CRY) that are transactivated by the positive arm CLOCK : BMAL1. Cellular accumulation of translated PER and CRY proteins reaches a peak during the biological active phase, resulting in the nuclear translocation where the circadian repressors interact with CLOCK and BMAL1 to repress their own transcription. PER and CRY are targeted by the E3 ubiquitin ligase and subsequently degraded by the proteasome, which allows CLOCK and BMAL1 to re-play a new daily cycle of transactivation. Additional feedback loops are also involved in regulating the core circadian network: CLOCK : BMAL1 heterodimers transactivates nuclear receptor *Rev-erb* and retinoic acid-related orphan receptor (*Ror*). REV-ERB represses transcription of *Clock* and *Bmal1*, while ROR activates transcription of *Clock* and *Bmal1*. The clock system is precisely regulated by multiple layers of transcriptional and translational mechanisms. Importantly, the E-box element that CLOCK and BMAL1 binds appears in a broad variety of gene promoters and enhancers, including genes encoding metabolic enzymes. Therefore, in addition to the clock components, thousands of genes are directly targeted by clock proteins, resulting in a robust oscillation of temporal gene expression and protein expression. That is how the circadian clock precisely controls biological rhythms at molecular levels.

The experiments studying the implication of the circadian clock into biological functions including innate immune systems as well as macrophage functions have been performed in a variety of biological systems but most frequently in small rodents. Mice and rats share many common genetic, physiological, and behavioral signatures with humans, many of which exhibit temporal dynamics in a circadian fashion. The crucial differences in the circadian clock system and circadian regulation of biological functions between humans and small rodents are diurnal and nocturnal, respectively. Temporal signatures of molecular rhythms and physiological activities in small rodents exhibit antiphase against diurnal animals (Mure et al., 2018). For instance, the light phase of small rodents corresponds to the rest phase when the positive arm (CLOCK and BMAL1) within the TTFLs drives gene transcription, whilst the dark phase of small rodents corresponds to the active phase when the negative arm (PER and CRY) represses the activity of CLOCK and BMAL1, which are opposite in diurnal animals such as humans. Excluding the difference in the phase of molecular clock activity and subsequent temporal characterization of physiology and behavior, small rodents are useful experimental materials to study how the circadian clock governs macrophage functions. Experimental small rodents are housed under temperature- and light/dark cycle-controlled environments. Most animal experiments have been done under 12h light/dark cycles, considering natural light/dark cycles. Since light is the dominant external time cue (*zeitgeber*), the timing when the light turns on and off is defined as zeitgeber time (ZT) 0 and ZT12, respectively. Essentially, ZT0-12 and ZT12-24 are considered as the rest phase and the active phase in nocturnal animals, respectively. Accordingly, small rodents serve as a comparative model of human circadian biological studies. The basic guideline of how to perform circadian animal experiments is available elsewhere (Eckel-Mahan and Sassone-Corsi, 2015).

## Circadian Clock Regulation of Inflammatory Functions of Macrophages

A series of inflammatory cytokines exhibit robust daily rhythms at the transcriptional and secretory levels (Gibbs et al., 2012). The response of gene expression as well as secretion of inflammatory cytokines including interleukin-6 (IL-6), IL-12, C-C-motif chemokine ligand 2 (CCL2), CCL5, and C-X-C-motif chemokine ligand 1 (CXCL1) to LPS stimulation in peritoneal exudate cells (PECs), which are predominantly macrophage, is dependent on the daily timing of LPS stimulation. The time-of-day variation of inflammatory cytokines in response to LPS stimulation is eliminated in the mice lacking circadian molecular rhythms. The induction of inflammatory cytokines is suppressed by the pharmacological activation of the circadian repressor REV-ERBα, indicating the circadian clock REV-ERBα mediates the regulation of inflammatory cytokines. Whether REV-ERBα directly or indirectly modulates the expression of inflammatory cytokines has been an outstanding question to accumulate molecular insight into the circadian clock regulation of inflammatory functions. Using pharmacological and genetic targeting in murine macrophages, the author’s group previously revealed that REV-ERBα is a direct repressor of *Ccl2* (also known as monocyte chemoattractant protein-1 (MCP-1)) gene (Sato et al., 2014b). REV-ERBα is found to directly bind a ROR response element at the promoter region of *Ccl2* gene. Consequently, REV-ERBα suppresses CCL2-activated intracellular mitogen-activated protein kinase (MAPK) pathways, including extracellular signal-regulated kinase (ERK) and p38, resulting in impaired cell adhesion and migration of macrophages. In contrast, RORα transactivates *Ccl2* gene *via* a direct interaction with *Ccl2* promoter and consequently, thereby activating the downstream pathways. Furthermore, mice lacking *Rev-erbα* display increases in *Ccl2* gene expression in peritoneal macrophages. In addition to *Ccl2* gene, *Il-6* gene is also identified not only as a direct target of REV-ERBα and RORα but also as an indirect target of REV-ERBα and RORα through modulation of nuclear factor-kappa B (NF-κB) activity (Sato et al., 2014a). Thus, transcriptional regulation of inflammatory cytokines by the circadian clock REV-ERBα and RORα represents the molecular mechanism by which the circadian clock orchestrates innate immunity and points to the link between impaired clockwork and chronic inflammation. Moreover, an investigation of the implication of the circadian clock into pattern recognition receptors (PRRs) leads to the discovery of how the circadian clock controls toll-like receptor (TLR) 9-mediated innate and adaptive immunity of macrophages (Silver et al., 2012). Direct binding of circadian transcription factor CLOCK : BMAL1 is essential for circadian gene expression of *Tlr9* in peritoneal macrophages. Strikingly, the response to TLR9 ligand exhibits robust time-of-day variation: 1) the time-of-day determines diseases severity in a TLR9-dependent sepsis model, and 2) timing of immunization determines TLR9 ligand-adjuvanted vaccine responsiveness. These findings demonstrated the biological molecular clock-driven circadian oscillation of immune functions of macrophages.

## Circadian Control of Inflammatory Functions Through Neuroendocrine Mechanisms

Circadian regulation of the sympathetic nervous system and endocrine secretion results in temporal dynamics of immune responses not only in macrophages but also broad immune cells (**Figure 1**). Of note, adrenergic systems, including the catecholamine neurotransmitter adrenaline and noradrenaline and hormonal mechanisms through the hypothalamic-pituitary-adrenal-axis (HPA axis), receive inputs from the external environment as well as internal biological clock machinery and send signals to immune cells, including macrophages (Nakai et al., 2014; Leach and Suzuki, 2020). Specifically, β2-adrenergic G-protein-coupled receptor-mediated signals play a key role in the circadian regulation of the adaptive immune response by modulating diurnal lymphocyte recirculation through lymph nodes. Since β2-adrenergic receptor regulates the innate immune response of macrophages by modulating TLR4 signaling (Kizaki et al., 2008; Kizaki et al., 2009), β2-adrenergic receptor signaling may coordinate circadian variation of macrophage functions. In relation to the role played by G-protein-coupled receptors in the circadian regulation of immune functions, circadian repressors CRY1/2 suppresses the adenylyl cyclase activity and cAMP generation, thereby inhibiting NF-κB activity through dephosphorylation of NF-κB p65 at serine 276 in macrophages (Narasimamurthy et al., 2012).

Another line of evidence is the intertwined link between glucocorticoid and immune activity (Shimba and Ikuta, 2020). Glucocorticoid is known as an ‘awake hormone’ and blood circulatory glucocorticoid levels oscillate with a peak in the early morning and trough at night in humans. Light-dark cycles are the critical regulator of circadian glucocorticoid secretion. Light stimuli are transmitted to the SCN in the hypothalamic region where secrets corticotropin-releasing hormone (CRH). CRH stimulates the anterior pituitary, leading to the secretion of adrenocorticotropic hormone (ACTH) which then stimulates the adrenal cortex to produce glucocorticoids. Glucocorticoids bind to glucocorticoid receptor ubiquitously expressed in most cells throughout the body, resulting in a broad range of physiological responses (Huang et al., 2010). The role of glucocorticoid signals in reprogramming the circadian molecular clock machinery has been well established. Glucocorticoid and the receptor complex directly interact with the promoter of clock genes, such as *Per1* and *Per2*, and regulate the circadian gene expression (Torra et al., 2000; So et al., 2009; Cheon et al., 2013). On the other hand, the circadian repressor CRY1/2 interacts with glucocorticoid receptors and alters the transcriptional response to glucocorticoids (Lamia et al., 2011). Glucocorticoid exerts strong anti-inflammatory and immunosuppressive effects and thereby are widely used for the treatment of inflammatory and autoimmune diseases (Farrell and Kelleher, 2003). The robust circadian oscillation of circulatory glucocorticoid results in the circadian regulation of inflammatory cytokine and chemokine expression, which governs the cyclic activity of systemic inflammation and immune response. For instance, CXCL5 is identified as a key molecule linking the circadian clock machinery in pulmonary epithelial cells to glucocorticoid action (Gibbs et al., 2014). In addition, glucocorticoid induces T cell migration into the spleen from blood and enhances immune response during the biological active phase through IL-7R and CXCR4 (Shimba et al., 2018). Glucocorticoid also shifts the phenotype of macrophages (van de Garde et al., 2014), indicating temporal remodeling of macrophage polarization and functions through the glucocorticoid-dependent mechanisms.

## Metabolism and Circadian Timing: Determinants of Macrophage Functions

Chronic and systemic inflammation is closely linked to aging and obesity and is a potent contributor to metabolic diseases, cardiovascular diseases, neurodegenerative diseases, musculoskeletal disorders, and cancer (Freund et al., 2010; Osborn and Olefsky, 2012). Chronically inflamed tissues are characterized by the presence of infiltration of inflammatory cells, such as macrophages (Nathan, 2002). Recent evidence accumulated during the past decade has revealed how pathogenic and inflammatory signals drive macrophage differentiation toward “attacking” phenotype by modulating macrophage signaling pathways and consequent gene programs (Galván-Peña and O’Neill, 2014; Kelly and O’Neill, 2015; Jing et al., 2020; Liu et al., 2021). Besides pathogenic and inflammatory signals, nutritional challenges and nutrient signals also reprogram macrophage functions (Olefsky and Glass, 2010). Specifically, free fatty acids derived from adipocytes and hepatocytes act as ligands against TLR4 and activate macrophages leading to the infiltration of activated macrophages into metabolic tissues (Shi et al., 2006; Nguyen et al., 2007). In support of this notion, physical exercise reverts the high-fat diet-induced inflammatory phenotype of macrophages (Kizaki et al., 2011). Since the daily timing has been demonstrated as a critical variable of metabolic outcomes from food intake and physical exercise (Asher and Sassone-Corsi, 2015; Sato et al., 2019), macrophage functions can be further optimized through rewiring of metabolic pathways at local and systemic levels following when to eat and exercise. In that sense, chronobiology-based behavioral regimens and chrono-nutritional regimens might be effective to sustain anti-inflammatory macrophages, and subsequently, bring a myriad of health benefits. Time-restricted eating is a well-identified strategy aiming at modulating macrophage inflammatory functions mediating the modification of temporal eating behaviors.

In addition to what we eat and how much we eat, when we eat is a critical determinant for metabolic health (Vollmers et al., 2009; Hatori et al., 2012; Chaix et al., 2014; Zarrinpar et al., 2014; Asher and Sassone-Corsi, 2015; Gill et al., 2015; Longo and Panda, 2016; Zarrinpar et al., 2016; Manoogian and Panda, 2017; Melkani and Panda, 2017; Chaix et al., 2019a; Chaix et al., 2019b; Manoogian et al., 2019; Wilkinson et al., 2020; Chaix et al., 2021). Specifically, a time-restricted feeding regimen limiting food access only during the biological active phase without reducing caloric intake prevents high-fat diet-induced metabolic diseases and systemic inflammation. The elevation of macrophage inflammatory cytokine markers in white adipose tissue upon a high-fat diet feeding is disappeared under a time-restricted feeding regimen (Hatori et al., 2012). More critically, macrophage infiltration in response to high-fat diet feeding is dramatically suppressed by time-restricted feeding (Delahaye et al., 2018). Most importantly, time-restricted feeding, conceptionally equal to eating at the biological active time, alters innate immune activity and reduces inflammation both in mice and humans (Cissé et al., 2018; Moro et al., 2020). Thus, there is an indirect contribution of the circadian clock to macrophage functions: circadian clock regulation of metabolic homeostasis characterizes inflammatory signatures of macrophages.

Next we summarize below the latest evidence of how macrophages act against specific pathogenic infections, and how the circadian clock modulates the role played by macrophages in the immune responses.

## Major Causes of Acute Lower Respiratory Tract Infections (LRTIs): Risk of Circadian Rhythm Disturbances

According to estimates by the World Health Organization (WHO) in 2019, among communicable diseases, acute LRTIs are the deadliest and killing 2.6 million people around the world, making it the fourth leading cause of death (WHO, 2020). Pneumonia is the major cause of death since it causes respiratory failure by filling the alveoli with fluids and pus that result from inflammation (WHO, 2019). Pathogenic microorganisms that develop pneumonia include bacteria, viruses, and fungi. Among these, *S. pneumoniae* is the most frequent bacterial cause of pneumonia. Indeed, in all generations, *S. pneumoniae* accounted for around 50% of the pathogens that caused deaths, contributing to far more deaths than all other major etiologies combined (respiratory syncytial virus, *H. influenzae* type b, and influenza) (GBD 2016 Lower Respiratory Infections Collaborators, 2018). Seasonal influenza is epidemic every winter, resulting annually in 290,000 to 500,000 deaths from respiratory illness (WHO, 2018). In particular, influenza A viruses have a high mutagenic capacity to generate new strains that can escape from acquired immunity, which has led to a pandemic every few decades. The ongoing pandemic is coronavirus disease 2019 (COVID-19) caused by severe acute respiratory syndrome coronavirus 2 (SARS-CoV-2). According to worldwide monitoring by the WHO, as of January 2022, confirmed cases of deaths have risen to more than 5 million (WHO, 2021). Remarkably, shift workers are reported to be at a higher risk of developing COVID-19 (Fatima et al., 2021; Maidstone et al., 2021). Even before the COVID-19 pandemic, shift work has been closely associated with the incidence and severity of influenza-like illness and acute respiratory infection (Loef et al., 2019; Loef et al., 2020). Accordingly, the disruption of circadian rhythms is more likely to suffer severe illness by exacerbating the replication or proliferation of pathogens and resultant pneumonia.

Alveolar macrophages are at the forefront of defense against LRTIs and prevent pneumonia by eliminating both airborne pathogens and dead cells through phagocytosis, pro-inflammatory responses, and efferocytosis. Since it has begun to be uncovered that these macrophage functions are precisely adjusted by the circadian clock, increased susceptibility to acute LRTIs and pneumonia due to circadian misalignment can be supposed to be partly caused by macrophage dysfunction.

## Role of Macrophage Phagocytosis in Acute LRTIs: Implication of the Circadian Clock

**Figure 2:**
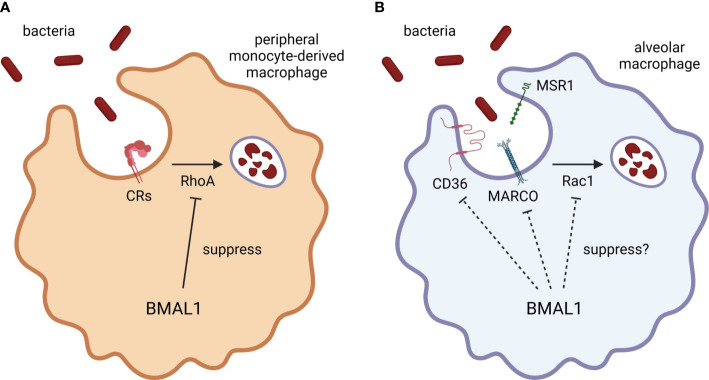
BMAL1 regulation of macrophage phagocytosis. BMAL1 suppresses bacterial phagocytosis by inhibiting RhoA activation in monocyte/macrophage lineage. **(A)** CRs require RhoA activation to engulf the antigens. Since peripheral monocytes express CR3, the phagocytic activity of peripheral monocyte-derived macrophages may be strongly regulated by BMAL1. **(B)** Although the phagocytic activity of alveolar macrophages is also improved by BMAL1 deletion, BMAL1 may regulate the expression of scavenger receptors or other signaling components as alveolar macrophages hardly express CRs. Thus, misaligned circadian rhythms may disturb the autonomic control of macrophage phagocytosis by disrupting temporal variation of BMAL1 expression. CR, complement receptor; BMAL1, brain and muscle ARNT-like 1; MARCO, macrophage receptor with collagenous structure; MSR1, macrophage scavenger receptor 1; Rac1, Ras-related C3 botulinus toxin substrate 1; RhoA, Ras homolog family member A.

Lung-resident alveolar macrophages play a leading role through phagocytosis in the clearance of airborne microorganisms that enter the alveoli by inspiration. Alveolar macrophages constitutively express macrophage scavenger receptor 1 (MSR1), macrophage receptor with collagenous structure (MARCO), and CD36 which are essential to eliminate both Gram-positive and Gram-negative bacteria (Arredouani et al., 2004; Arredouani et al., 2005; Arredouani et al., 2006; Sharif et al., 2013) (**Figure 2**). After pathogen recognition by these pattern recognition receptors (PRRs), the engulfment of pathogens requires cytoskeleton remodeling, which is primarily controlled by three Ras homolog (Rho) family small-GTP binding proteins, including Rho family member A (RhoA), Ras-related C3 botulinus toxin substrate 1 (Rac1), and cell division control protein 42 homolog (Mao and Finnemann, 2015). Although the downstream pathways of MSR1 and CD36 have not yet been elucidated, a recent study showed that *E. coli* interacts with MARCO, which activates Rac1 to initiate filamentous (F)-actin polymerization, filopodia formation, and subsequent engulfment in murine alveolar macrophages (Li et al., 2017) (**Figure 2**). Notably, aged mice have a weaker expression of Rac1 and reduced phagocytosis (Li et al., 2017).

Several *ex vivo* studies revealed that the phagocytic activity of macrophages exhibits circadian rhythmicity. Phagocytosis of serum-opsonized zymosan of murine peritoneal exudate macrophages exhibits a circadian variation, whose activity reaches a daily peak during the resting period and a trough during the active period (Hayashi et al., 2007). Murine peritoneal macrophages also have circadian variation in phagocytosis of non-opsonized *S. typhimurium* (Oliva-Ramírez et al., 2014). Likewise, murine peritoneal resident macrophages elicit greater phagocytic activity against non-opsonized *E. coli* and *S. aureus* at the end of the resting period than at the end of the active period, with a similar expression pattern of circadian variation observed in cell surface CD36 expression, whereas circadian oscillations of macrophage phagocytosis are not reproduced using an *in vivo* intraperitoneal *S. aureus* infection model (Geiger et al., 2019). Given these results from a series of *ex vivo* experiments, both tissue-resident and peripheral monocyte-derived macrophages have similar circadian rhythmicity in the core clock gene expression, causing autonomic regulation of phagocytic activity. In particular, the activity augments during the resting period and oscillates in antiphase to diurnal variation in *Bmal1* gene expression.

The most recent study uncovered *in vivo* functional role of BMAL1 in the circadian rhythmicity of macrophage phagocytosis (**Figure 2**). Myeloid cells-selective BMAL1-deleted mice accelerate bacterial removal and diminish cytokine production in the lung after intranasal *S. pneumoniae* inoculation at the end of the resting period, which are mediated by enhanced phagocytosis through activation of RhoA in quiescent BMAL1-deficient macrophages (Kitchen et al., 2020). The enhanced phagocytosis by myeloid BMAL1 deletion is particularly pronounced in peritoneal exudate cells during intraperitoneal *S. aureus* infection and in peritoneal exudate and alveolar macrophages during *ex vivo S. aureus* challenge, while the effects are moderate and not significant in neutrophils, suggesting that BMAL1 suppresses bacterial phagocytosis by inhibiting RhoA activation especially in monocyte/macrophage lineage (Kitchen et al., 2020). However, there are the complement receptors that require RhoA activation to engulf the antigens (Caron and Hall, 1998). A previous study reported that murine alveolar macrophages hardly express complement receptors CR1/2/3 and their phagocytic activity is not enhanced by complement opsonization (Berger et al., 1994), whereas peripheral monocytes express CR3 (Hughes and Gordon, 1998) and bone marrow-derived macrophages differentiated with macrophage colony-stimulating factor produced pro-inflammatory cytokines in response to complement-opsonized antigens (Acharya et al., 2020). Therefore, it could be a reasonable hypothesis that the augmented phagocytosis in BMAL1-deleted alveolar macrophages is mediated by increased cell surface expression of phagocytic receptors and/or activation of other signaling molecules such as Rac1 rather than RhoA (**Figure 2**).

In contrast, another latest study comprehensively explored post-transcriptional and post-translational circadian regulation of macrophage functions and provided a novel insight into the underlying machinery of circadian regulation of phagocytosis. In fact, both transcriptome and proteome analyses in murine bone marrow-derived macrophages revealed that only about 15% of the circadian proteome have corresponding mRNA oscillations, suggesting that most of the proteins that exhibit circadian rhythm in macrophages are not directly regulated according to the central dogma (Collins et al., 2021). Moreover, most of enzymes involved in mitochondrial respiration, including complexes I, III, IV, and V, show circadian variability coupled with circadian phagocytic activity, and thus blocking mitochondrial respiration by oligomycin results in the disappearance of the circadian rhythmicity of macrophage phagocytosis against zymosan (Collins et al., 2021). Since the formation and polymerization of F-actin during engulfment requires a continuous supply of ATP, circadian metabolic regulation could largely influence the phagocytic activity of either alveolar or monocyte-derived macrophages.

The mounting evidence strongly indicates that the timing of bacterial inhalation determines whether macrophages can phagocytize and eliminate them effectively. Especially during the resting period, the ability to remove pathogens in the alveoli is supposed to be increased by enhanced phagocytic activity of both alveolar and peripheral monocyte-derived macrophages. Conversely, misalignment of circadian rhythms due to irregular lifestyle such as disrupted sleep-wake cycles may disturb the autonomic control of macrophage phagocytosis by impaired temporal oscillation of core clock gene expression. As described above, macrophage phagocytosis requires not only sufficient expression of phagocytic receptors that recognize pathogens but also the coordinated action of intracellular molecules mediating subsequent engulfment. It should also be noted that the expression patterns of these receptors vary widely depending on the microenvironment where macrophages are differentiated, as has already been explored (Berger et al., 1994; A-Gonzalez et al., 2017; Bruggeman et al., 2019). Moreover, phagocytosis is affected by circadian metabolic regulation. It is therefore expected that the expression and activity of molecules related to phagocytosis as well as post-transcriptional and post-translational regulation will be systematically considered for each cell type to elucidate the whole picture of the circadian control of macrophage phagocytosis.

## Role of Macrophage Pro-Inflammatory Responses in Acute LRTIs: Implication of the Circadian Clock

**Figure 3:**
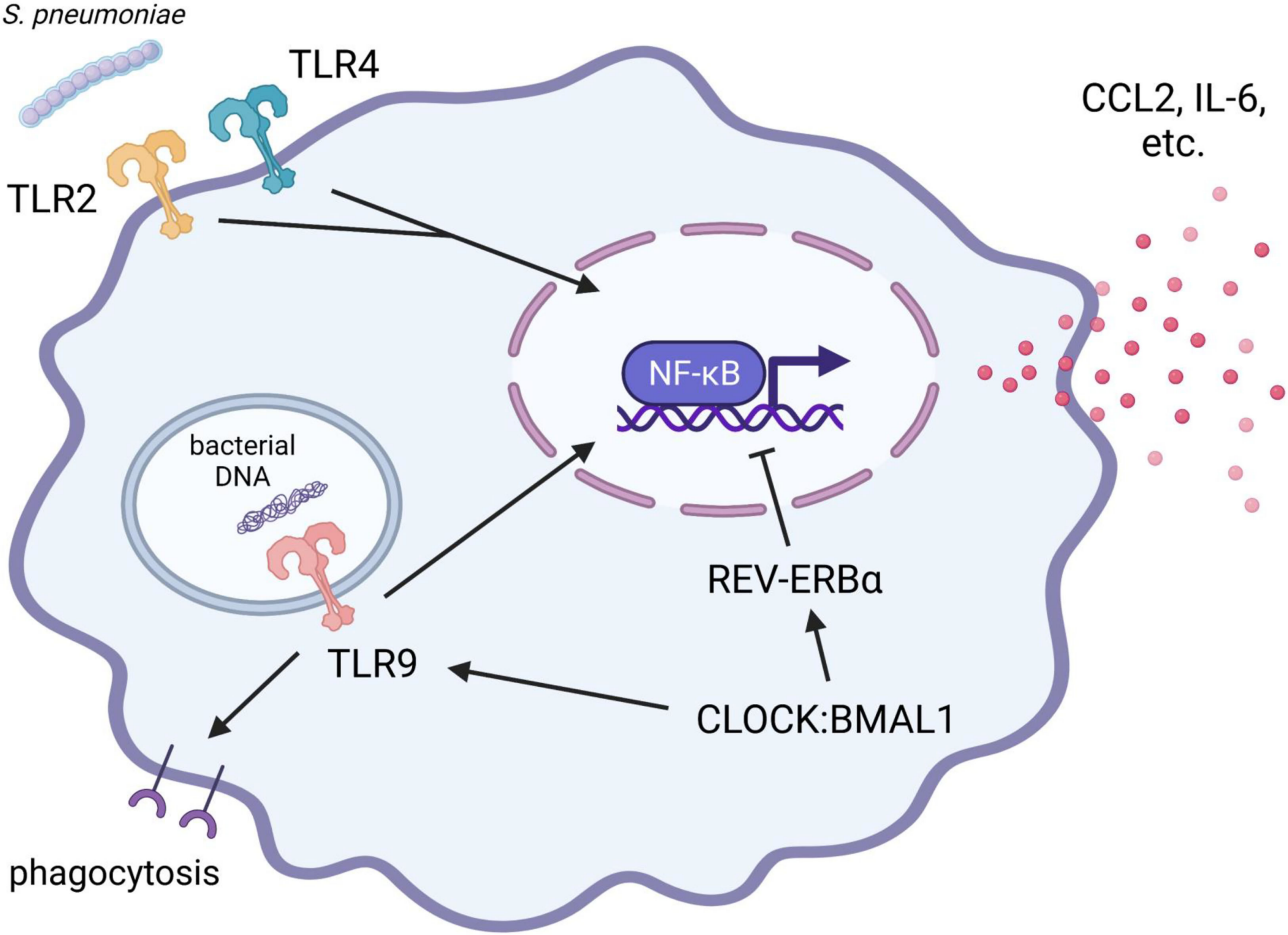
Protective roles of TLR2, TLR4, and TLR9 during pulmonary *S. pneumoniae* infection: implication of the circadian clock. The pro-inflammatory responses *via* TLR2 signaling play a supporting part in eliciting local inflammation and bactericidal activity against *S. pneumoniae*. In contrast, initial pro-inflammatory responses *via* TLR4 signaling in alveolar macrophages contribute to the prompt elimination of *S. pneumoniae* and the prevention of life-threatening pneumonia. REV-ERBα suppresses the production of pro-inflammatory cytokines (such as IL-6) and chemokines (such as CCL2). TLR9 signaling is indispensable for upregulating the phagocytic activity of alveolar macrophages to prevent systemic transmission of *S. pneumoniae*. The expression and function of TLR9 are positively regulated by the CLOCK : BMAL1 heterodimer. Therefore, since the pro-inflammatory responses of alveolar macrophages are complexly and precisely controlled by clock components, the disruption of circadian rhythms may lead to diminished ability to eliminate bacteria and consequent exacerbation of pneumonia. BMAL1, brain and muscle ARNT-like 1; CCL2, C-C motif chemokine ligand 2; CLOCK, Circadian locomotor output cycles kaput; IL-6, interleukin 6; TLR, toll-like receptor.

In addition to phagocytosis, alveolar macrophages provoke pro-inflammatory responses by detecting pathogen-associated molecular patterns using a wide variety of PRRs, including TLRs, to facilitate immediate mobilization and activation of phagocytes such as neutrophils and monocytes. The protective role of TLR2, TLR4, and TLR9 against pneumococcal infection is suggested by the results obtained from studies using either TLR2, TLR4, or TLR9 knockout or mutant mice (Branger et al., 2004; Knapp et al., 2004; Albiger et al., 2007) (**Figure 3**). In particular, TLR4 mutant mice show deteriorated survival rates after inoculation of a non-lethal dose of *S. pneumoniae*, accompanied by increased bacterial growth, monocyte and lymphocyte infiltration, and interstitial inflammation in the lung (Branger et al., 2004). TLR9 knockout mice develop pulmonary inflammation during *S. pneumoniae* infection in accordance with wild-type mice, but show worse survival and more bacterial invasion from the bronchoalveolar fluids into lung tissue and bloodstream, with abrogated upregulation of phagocytic activity in alveolar macrophages (Albiger et al., 2007). A recent study also demonstrated that, although mice lacking TLR4 exhibit lower viability and augmented colonization in the lung after intranasal *S. pneumoniae* inoculation compared to wild-type mice, this exacerbation of infection is accompanied by attenuated pro-inflammatory profile, reduced live alveolar macrophages, diminished infiltration of neutrophils and monocytes, and inhibited differentiation from monocyte into macrophages (Sánchez-Tarjuelo et al., 2020).

Compared to phagocytosis, circadian clock regulation of pro-inflammatory responses in monocyte/macrophage lineage is more deeply understood (Timmons et al., 2020). As discussed earlier in this review, LPS-induced production of pro-inflammatory cytokines and chemokines by murine peritoneal exudate macrophages shows circadian gating as observed in phagocytosis. The circadian gating and rhythmic expression of REV-ERBα are abolished by myeloid-selective BMAL1 deletion (Gibbs et al., 2012). Moreover, REV-REBα agonist broadly suppresses LPS-stimulated gene expression of pro-inflammatory mediators (Gibbs et al., 2012), and in particular, REV-ERBα is found to repress CCL2 expression by interacting directly with the promoter region (Sato et al., 2014b). REV-ERBα also suppresses LPS-stimulated IL-6 expression in both direct and indirect manners (Sato et al., 2014a). In the human primary cell culture model, the suppression by REV-ERBα is more prominent in alveolar macrophages than in peripheral monocyte-derived macrophages (Gibbs et al., 2012). LPS-stimulated pro-inflammatory responses are also heightened in alveolar macrophages isolated from mice lacking REV-ERBα compared to wild-type mice (Pariollaud et al., 2018). These findings suggest that the core clock component BMAL1 exerts anti-inflammatory effects *via* REV-ERBα in both alveolar and peripheral monocyte-derived macrophages (**Figure 3**).

Furthermore, myeloid-selective BMAL1 knockout mice exhibit amplification of LPS-induced NF-κB phosphorylation in peritoneal exudate macrophages isolated at the end of the active period when *Bmal1* gene expression reaches a daily peak (Curtis et al., 2015), suggesting that BMAL1 interferes with TLR4 signaling. However, simultaneously, LPS stimulation induces miRNA-155, which provides positive feedback to improve TLR4 signaling by degrading BMAL1 (Curtis et al., 2015). Thus, during bacterial infection, macrophages may be reprogrammed to adequately elicit initial pro-inflammatory responses and phagocytosis by temporarily canceling the anti-inflammatory effects of BMAL1. As another possible mechanism by which pro-inflammatory stimuli can temporarily reprogram macrophage circadian clocks, a variety of diverse agonists such as interferon γ (IFN-γ), tumor necrosis factor-α, LPS, and Pam3CSK4 (TLR2/1 heterodimer agonist) dampens serum shock-induced oscillation of *Per2* and *Rev-erbα* transcripts in different manners in murine bone marrow-derived and peritoneal exudate macrophages (Chen et al., 2020). This suggests that pro-inflammatory agonists provide positive feedback to improve downstream signaling of their receptors by attenuating the oscillations of these negative-arm clock components in macrophages.

An *in vivo* study has addressed the impact of lethal systemic endotoxemia on the reprogramming of circadian rhythms in the lung. During the two days from the onset of endotoxemia at the late resting phase to death, the circadian expression rhythm of the *Bmal1* gene becomes arrhythmic and remains at a high level in the lung, while that of the *Rev-erbα* gene shows a distorted but rhythmic pattern compared to healthy lungs (Haspel et al., 2014). The number of neutrophils exhibits lung-specific diurnal variation with larger amplitude, which remains rhythmic also during endotoxemia, but the mobilization and circadian pattern disappeared in *Bmal1*-deleted lungs (Haspel et al., 2014). Notably, alveolar macrophages exhibit slight but significant chronotypes in healthy lungs. Since alveolar macrophages are self-maintained by the paracrine action of granulocyte-macrophage colony-stimulating factor secreted by epithelial cells (Schneider et al., 2014b), the maintenance of alveolar macrophages could be circadian regulated. Although the reprogramming manner observed *in vivo* and *in vitro* findings is likely to differ due to complicated interactions with tissue structure cells, it is expected to elucidate the *in vivo* circadian reprogramming in the functions of alveolar and monocyte-derived macrophages during pulmonary infection.

It has also been reported that BMAL1 determines the extent of local inflammation through the regulation of peripheral inflammatory monocyte trafficking. The number of inflammatory monocytes in circulating blood displays circadian rhythmicity, with a daily peak during the resting period and a daily through during the active period, and in fact, the efficiency of inflammatory monocyte infiltration, bacterial removal, and pro-inflammatory responses after peritoneal inoculation with *L. monocytogenes* is higher at the middle of the resting period ZT 8 than at the beginning of the resting period (ZT 0) (Nguyen et al., 2013). However, myeloid-selective BMAL1 deletion abolishes this rhythmicity and constantly changes the inflammatory monocyte count to a high state by canceling the suppression of CCL2 expression, resulting in exacerbated inflammation and worse survival after peritoneal infection (Nguyen et al., 2013). In addition, the REV-ERB isoforms repress the expression of the *Mmp9* and *Cxc3cr1* genes, which regulate cellular infiltration and migration, by inhibiting the transcription of their non-coding RNAs that act as enhancers in murine macrophage lineages, including bone marrow-derived and peritoneal exudate macrophages (Lam et al., 2013), strongly indicating that the degree of monocyte infiltration during pulmonary infection and the resulting pathogen clearance and/or pneumonia are also controlled by the circadian clock. On the other hand, regarding pulmonary fibrosis, REV-ERBα in fibroblasts but not in myeloid cells serve a major role in the development (Cunningham et al., 2020). Thus, the function of REV-ERBα is cell-selective, and the disruption of its rhythmicity leads to different disorders.

For DNA viruses, the BMAL1:CLOCK heterodimer exerts anti-viral activity without modification of the TLR signaling. That is, REV-ERBα inhibits hepatitis B virus replication by interacting with the E-box motifs of viral DNA (Zhuang et al., 2021). In terms of double-stranded RNA (dsRNA) viruses such as influenza A virus and SARS-CoV-2, the dsRNA replicated intracellularly after infection is detected by endosomal TLR3, which drives the production of type I IFNs (Kawasaki and Kawai, 2014). As IFN receptors are ubiquitously expressed, IFN-α/β act on both infected and neighboring cells to induce the expression of hundreds of IFN-stimulated genes, including products that inhibit viral replication. In murine spleen, TLR3 protein levels and responsiveness to its ligand poly(I:C) exhibit time-of-day specificity, albeit with being not observed in splenic macrophages (Silver et al., 2018). Taking COVID-19 as an example, it has been under argument whether the initial type I IFN responses mediated by TLR3 are sufficiently involved in the onset and aggravation of COVID-19 or not. For instance, people with a mutation of TLR3 or IFN-α receptor (Zhang et al., 2020b) and with autoantibody against IFN-α (Bastard et al., 2020) have a higher risk of COVID-19 exacerbation. SARS-CoV-2 proteins ORF-3b and ORF-6 have also been identified to suppress type I IFN induction (Konno et al., 2020; Kimura et al., 2021). Therefore, elucidating the circadian clock regulation of TLR3 signaling in the alveolar microenvironment is crucial in exploring mechanisms by which the disruption of the circadian rhythms increases the risk of developing COVID-19.

Moreover, the effects of an inactivated vaccine against SARS-CoV-2, including neutralizing antibody production, B lymphocyte and follicular helper T lymphocyte responsiveness, monocyte and dendritic cell infiltration, and memory B lymphocyte count, are reported to be higher when inoculated in the morning than in the afternoon (Barnoud et al., 2021; Zhang et al., 2021). According to the recent findings, envelop protein, spike protein, and RNA derived from SARS-CoV-2 were sensed by TLR2 (Zheng et al., 2021), TLR4 (Shirato and Kizaki, 2021; Zhao et al., 2021), and TLR3/7 (Bortolotti et al., 2021; Salvi et al., 2021), respectively. It is presumed that the circadian control of TLR signaling in tissue-resident macrophages specify the time window of vaccine effect.

## Role of Macrophage Efferocytosis in Acute LRTIs: Implication of the Circadian Clock

**Figure 4:**
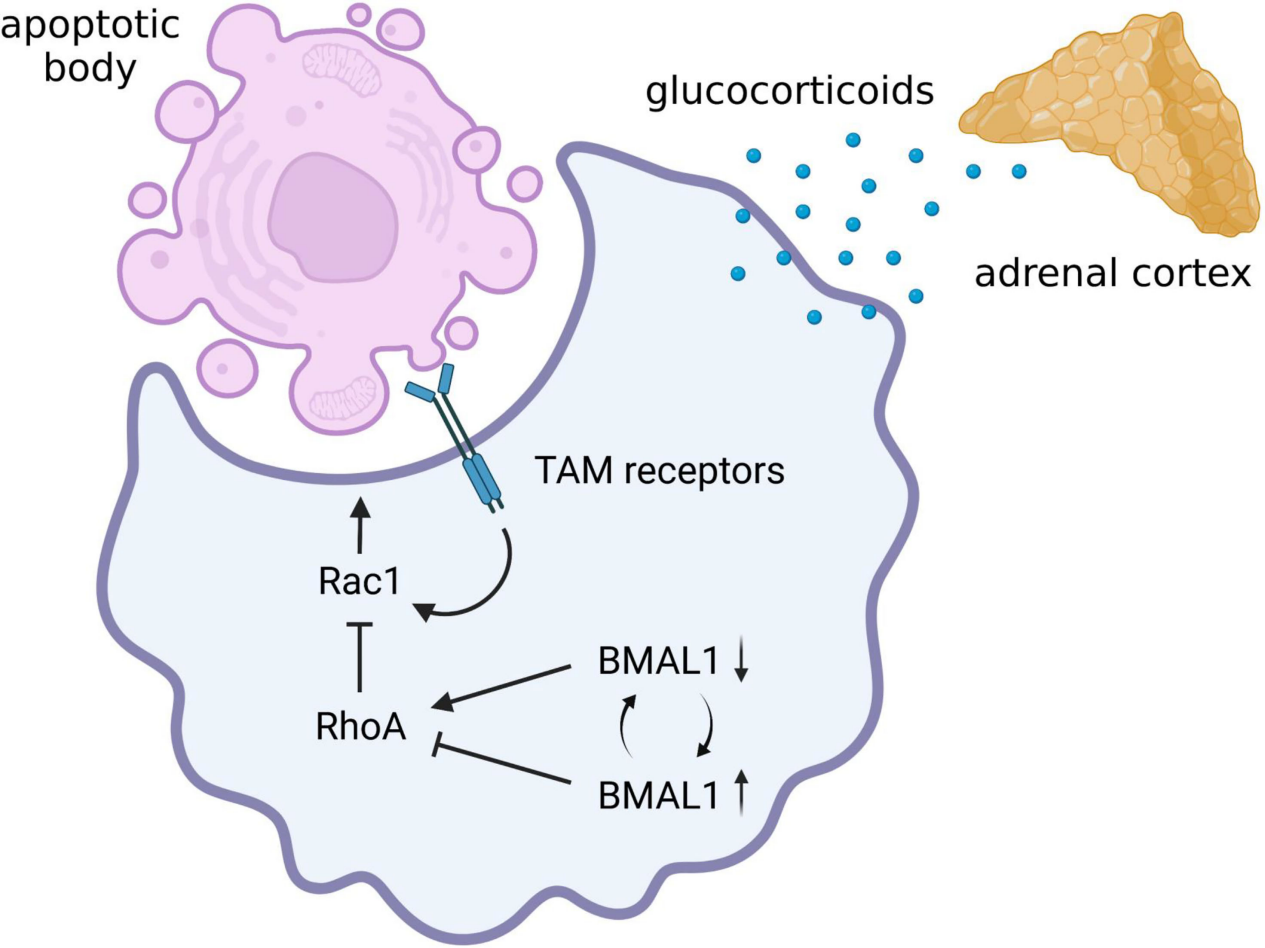
Possible circadian regulation of efferocytosis by alveolar macrophage. Alveolar macrophages have remarkable efferocytosis activity mediated by the TAM receptors. There is still no evidence of whether the efferocytosis activity of alveolar macrophages is circadian regulated. However, augmented activation of RhoA due to BMAL1 deprivation can lead to a marked decrease in efferocytosis activity of alveolar macrophages by inhibiting Rac1 activity. In addition, since glucocorticoids augment efferocytosis, macrophage efferocytosis may also be influenced by circadian rhythmicity of hypothalamic-pituitary-adrenal (HPA) axis activity. Therefore, there is a possibility that the immunomodulatory effects of intracellular clock genes and the diurnal oscillation of sympathetic-pituitary-adrenal axis activity determine the severity of pneumonia during virus infection by controlling the clearance of apoptotic bodies. BMAL1, brain and muscle ARNT-like 1; Rac1, Ras-related C3 botulinus toxin substrate 1; RhoA, Ras homolog family member A; TAM, Tyro 3, Axl, and proto-oncogene c-mer tyrosine kinase.

Alveolar macrophage-depleted mice show severe manifestations after pulmonary infection with a non-lethal dose of influenza A virus, despite the fact that virus clearance is not largely impaired, which indicates their contribution to host survival *via* suppressing excessive pulmonary inflammation by removing endogenous apoptotic cells (termed efferocytosis) (Schneider et al., 2014a). Macrophages perform efferocytosis primarily using the tyrosine receptor kinases Tyro 3, Axl, and proto-oncogene c-mer tyrosine kinase (MerTK) (collectively abbreviated as TAM) as phosphatidylserine receptors (Seitz et al., 2007) (**Figure 4**). Murine alveolar macrophages highly express Axl and MerTK, but little or no expressions in lung-mobilized monocytes (Mohning et al., 2018). Human alveolar macrophages also predominantly express Axl and peripheral monocytes do not express either Axl or MerTK (Grabiec et al., 2017). Although Axl knockout mice manifest no inflammatory disorders under healthy condition, they show exaggerated severity during pulmonary infection with influenza A virus, accompanied by increased accumulation of apoptotic cells, elevated infiltration of neutrophils and T-lymphocytes, and promoted secretion of pro-inflammatory cytokines and chemokines, while virus clearance is not compromised (Fujimori et al., 2015). Similar to pathogen recognition by phagocytic receptors like MARCO, the ligation of TAM receptors results in activating Rac1, leading to membrane ruffling to engulf apoptotic bodies (Todt et al., 2004; Wu et al., 2005) (**Figure 4**).

Circadian rhythms of macrophage efferocytosis are observed in a series of studies exploring the mechanism of leukocyte turnover. It has long been known that the number of various leukocytes in circulating blood shows diurnal variation, and that of circulating lymphocyte exhibited a phase-delay following altered plasma corticosterone levels in *Clock* mutant mice (Oishi et al., 2006). The absolute count of circulating neutrophils also fluctuates in a circadian fusion, with the active release of young neutrophils between ZT 17 to ZT 5 and clearance of aged neutrophils between ZT 5 and ZT 13 in mice (Casanova-Acebes et al., 2013). In particular, circulating neutrophils are replaced into young neutrophils within a new daily cycle starting from ZT 17 while aged neutrophils virtually disappear from the blood. *MerTK* transcript in the bone marrow shows a circadian fluctuation in antiphase with the regenerative activity of circulating neutrophils, whereas this fluctuation is accompanied by enhanced transcriptional activity of the liver X receptor in bone marrow-resident macrophages that engulfed accumulated aged neutrophils (Casanova-Acebes et al., 2013). That is, in the case of aged neutrophil clearance in the bone marrow, efferocytosis activity of resident macrophage is regulated by diurnal turnover of neutrophils rather than the intracellular circadian clock genes.

Another outstanding experimental model is capable of live cell monitoring of endogenous cell clearance activity of tissue-resident macrophages by conjoining the blood circulation of CD45.2 DsRedTg (circulating leukocytes fluorescently tagged) mice to their partner non-fluorescence CD45.1 mice. This study revealed the proportion of macrophages that engulfed fluorescent leukocytes is higher at the end of the resting period (ZT 11) than in the beginning (ZT 3) in a wide range of tissues including the bone marrow, spleen, intestine, and lung interstitium of the partner mice (A-Gonzalez et al., 2017). Unfortunately, no activity in alveolar macrophages is observed in this model (A-Gonzalez et al., 2017), since alveolar macrophages are not replenished by peripheral progenitor cells after the fetal period (Schneider et al., 2014b) and leukocytes do not infiltrate into the alveoli unless infected or toxic. The circadian gating of macrophage clearance activity is similar to that of macrophage phagocytosis of bacteria (Hayashi et al., 2007; Geiger et al., 2019; Kitchen et al., 2020), indicating the possibility that the clearance activity for aged cell may be also dependent on core clock components such as BMAL1 in tissue-resident macrophages. However, although apoptosis has traditionally been thought to be the predominant form of cell death involved in homeostatic cellular turnover (Nagata et al., 2010), little is known whether fluorescent leukocytes engulfed by tissue-resident macrophages caused apoptotic cell death. Thus, it is still uncertain whether the endogenous cell clearance ability observed in this study reflects efferocytosis activity.

As already introduced in the above section, BMAL1 deletion in macrophages increases RhoA activity, leading to the improvement of phagocytosis against bacteria (Kitchen et al., 2020). Since RhoA activity is indispensable for complement receptor-mediated phagocytosis (Caron and Hall, 1998), the immunomodulatory effect may seem to be beneficial for bacterial removal from the alveoli. However, it has been hypothesized that RhoA has the opposite role to Rac1 in efferocytosis (Nakaya et al., 2006). Indeed, the decrease in efferocytosis activity due to abnormally increased RhoA expression is ameliorated by pharmacological inhibition of RhoA (Vandivier et al., 2009). Therefore, augmented activation of RhoA due to BMAL1 deficiency can lead to a marked decrease in efferocytosis activity in macrophages, which may increase the risk of exacerbating pneumonia during viral infections (**Figure 4**). In addition, glucocorticoids have long been known to be the hormonal factors that not only suppress the pro-inflammatory signaling but also promote macrophage efferocytosis (Giles et al., 2001). As plasma glucocorticoid levels exhibit circadian fluctuations (Oishi et al., 2006; Cuesta et al., 2017), macrophage efferocytosis may also be influenced by circadian rhythmicity of HPA axis activity (**Figure 4**). To date, there is still no direct evidence of whether the efferocytosis activity of alveolar macrophages is circadian regulated. Based on the latest insight, however, there is a possibility that the immunomodulatory effects of molecular clock components and the diurnal oscillation of HPA axis activity may determine the severity of pneumonia during virus infection by controlling the clearance of apoptotic bodies.

## Conclusion

Since 2019, the COVID-19 pandemic has been a world-wide health problem and torturing us. The coronavirus initiates pneumonia or systemic inflammatory responses and macrophages play essential roles in both the defense system and exacerbation process. This review summarizes how macrophages are activated through various pathogens and commit to the clearance of bacteria, fungi, and viruses, including coronavirus, especially focusing on the key functions such as phagocytosis, pro-inflammatory response, and efferocytosis. We also provide perspective on the implication of the time-of-day and the circadian clock into macrophage responses to pathogens, while carefully distinguishing between alveolar macrophages and peripheral monocyte-derived macrophages. The review not only paves the way for a better understanding of the role of macrophages in inflammatory responses but also generate a new hypothesis to determine how the time of day differentially specifies inflammatory responses of macrophages against acute LRTIs. We hope this review could help to end the battle with the pandemic, summarizing the evidence of time-for-macrophages in inflammatory responses.

## Author Contributions

KS and SS wrote the manuscript. All authors contributed to the article and approved the submitted version.

## Funding

SS is supported by the Brain & Behavioral Research Foundation (NARSAD Young Investigator Grant, 28681) and the start-up funds from Texas A&M University. KS is funded by a Grant-in-Aid for Scientific Research (C) (21K11472) from the Ministry of Education, Culture, Sports, Science and Technology, Japan and a Research Grant for Young Investigators from Kyorin University School of Medicine.

## Conflict of Interest

The authors declare that the research was conducted in the absence of any commercial or financial relationships that could be construed as a potential conflict of interest.

## Publisher’s Note

All claims expressed in this article are solely those of the authors and do not necessarily represent those of their affiliated organizations, or those of the publisher, the editors and the reviewers. Any product that may be evaluated in this article, or claim that may be made by its manufacturer, is not guaranteed or endorsed by the publisher.
